# Polymorphisms at Amino Acid Residues 141 and 154 Influence Conformational Variation in Ovine PrP

**DOI:** 10.1155/2014/372491

**Published:** 2014-07-14

**Authors:** Sujeong Yang, Alana M. Thackray, Lee Hopkins, Tom P. Monie, David F. Burke, Raymond Bujdoso

**Affiliations:** ^1^Department of Veterinary Medicine, University of Cambridge, Madingley Road, Cambridge CB3 OES, UK; ^2^Department of Zoology, University of Cambridge, Downing Street, Cambridge CB2 3EJ, UK

## Abstract

Polymorphisms in ovine PrP at amino acid residues 141 and 154 are associated with susceptibility to ovine prion disease: Leu141Arg154 with classical scrapie and Phe141Arg154 and Leu141His154 with atypical scrapie. Classical scrapie is naturally transmissible between sheep, whereas this may not be the case with atypical scrapie. Critical amino acid residues will determine the range or stability of structural changes within the ovine prion protein or its functional interaction with potential cofactors, during conversion of PrPC to PrPSc in these different forms of scrapie disease. Here we computationally identified that regions of ovine PrP, including those near amino acid residues 141 and 154, displayed more conservation than expected based on local structural environment. Molecular dynamics simulations showed these conserved regions of ovine PrP displayed genotypic differences in conformational repertoire and amino acid side-chain interactions. Significantly, Leu141Arg154 PrP adopted an extended beta sheet arrangement in the N-terminal palindromic region more frequently than the Phe141Arg154 and Leu141His154 variants. We supported these computational observations experimentally using circular dichroism spectroscopy and immunobiochemical studies on ovine recombinant PrP. Collectively, our observations show amino acid residues 141 and 154 influence secondary structure and conformational change in ovine PrP that may correlate with different forms of scrapie.

## 1. Introduction

Prion diseases, or transmissible spongiform encephalopathies (TSEs), are fatal neurodegenerative disorders that affect humans and other vertebrate species. These conditions include scrapie in sheep, bovine spongiform encephalopathy (BSE) in cattle, and Creutzfeldt-Jakob disease (CJD) of humans. Collectively, these diseases can manifest as inherited, infectious, or sporadic conditions [1]. A central event of prion pathogenesis is the structural conversion of the *α*-helix-rich host protein PrPC into an abnormal isomer PrPSc, characterised by an increase in *β*-sheet structure [2, 3]. PrPC is a copper binding [4, 5] cell-surface glycoprotein that comprises a relatively unstructured N-terminal domain and a predominantly globular C-terminal region containing three *α*-helices interdispersed by a short antiparallel *β*-sheet region [6–11]. The globular domain demonstrates a close association between helix-1, the C-terminal region of helix-2, and the N-terminal region of helix-3. This central core is bound by an intramolecular disulphide bond between amino acid residues in helix-2 and helix-3. Characterisation of the protein folding events that occur during the conformational change in PrP during prion disease is crucial to an understanding of the formation of PrPSc and its subsequent oligomerisation.

Scrapie disease of sheep is the prototypic prion disease. Four major polymorphisms in the ovine prion protein, located at amino acid residues 136, 141, 154, and 171, are associated, in some cases relatively [12, 13], with susceptibility to two classifications of scrapie disease [13–16]. Animals that express V^136^L^141^R^154^Q^171^ (VLRQ) or A^136^L^141^R^154^Q^171^ (ALRQ) ovine PrP are susceptible to classical scrapie, a transmissible form of ovine prion disease [17]. In contrast, atypical scrapie disease of sheep has been reported in classical scrapie-resistant PrP genotypes including A^136^L^141^R^154^R^171^ (ALRR), A^136^F^141^R^154^Q^171^ (AFRQ), and A^136^L^141^H^154^Q^171^ (ALHQ) [18]. Atypical scrapie disease usually occurs in old sheep and is not considered to be naturally transmissible. Epidemiological studies suggest that the ovine PrP allelic variants AFRQ and ALHQ are associated with the highest susceptibility to atypical scrapie disease [13] and that this condition is a spontaneous disorder of PrP folding and metabolism [18, 19], although natural transmission by oral exposure cannot yet be excluded [20].

Critical amino acid residues will determine the range or stability of structural changes within the ovine prion protein, or its functional interaction with potential cofactors, during conversion of PrPC to PrPSc. Computational techniques can be used to predict functional or critical structural amino acid residues within a specific protein [21–23]. The conservation of individual amino acids with a polypeptide sequence has been shown to be strongly dependent on the environment in which the residues occur in the protein structure [24, 25]. Therefore, the application of conservation of sequence analysis is able to distinguish between evolutionary restraints arising from the need to preserve protein function and those that arise from the preservation of the protein's structural environment. We have previously performed molecular dynamics simulations (*mds*) of the ovine PrP allelic variants VLRQ, ALRQ, and ALRR in order to determine how genotypic variation at amino and residues 136 and 171 influences conformational variation in ovine prion protein variants associated with susceptibility to classical scrapie [26]. Here we have used a combined computational and experimental approach using ALRQ, AFRQ, and ALHQ ovine PrP in order to determine how genotypic variation at amino acid residues 141 and 154 influences conformational variation in conserved regions of ovine prion protein variants associated with susceptibility to atypical scrapie.

Computationally, we have identified regions of the ovine PrP protein, some in close proximity to amino acid residues 141 and 154, which display a higher degree of conservation than would be expected on the basis of the local structural environment. These conserved regions of ovine PrP, which likely represent critical structural amino acid residues, showed genotypic differences in the range of structural conformations and amino acid side-chain interactions that could be adopted when analysed by* mds*. Significantly, an increase in *β*-sheet content involving amino acid residues 112–121 occurred most frequently in the ALRQ variant and least frequently in ALHQ. We have supported these computational observations with experimental studies. The propensity for sarkosyl- or copper-induced conformational change in ovine recombinant PrP, measured by circular dichroism (CD) spectroscopy and capture-detector ELISA, respectively, was in the order ALRQ ≥ AFRQ > ALHQ. Furthermore, amongst the three ovine PrP genotypes analysed, the ALRQ variant showed the highest propensity for aggregation. Collectively, these observations show that variants of ovine PrP display differences in secondary structure and conformational change. The data suggest a structural correlation for genotypic variants of ovine PrP and their association with different forms of scrapie.

## 2. Materials and Methods

### 2.1. Sequence Conservation Analysis

The Crescendo algorithm was used to identify amino acid substitutions that are likely to be involved in protein function or protein interactions [27]. This algorithm compares the observed sequence conservation for each amino acid position in the homologous sequences of a protein with the conservation pattern predicted on the basis of local environment substitution tables. Tables of the log-odds probability values of finding a given amino acid substitution, in a given structural environment, have previously been calculated for a variety of structural environments [24, 25]. Commonly used definitions of the structural environments include the accessibility of the side-chain of the amino acid to solvent; the conformation of the backbone of the amino acid (helix, strand, and coil); and whether the amino acid forms hydrogen bonds with other amino acids or ligands. These probability values represent the average substitution frequency of exchange of two amino acids in a given structural environment seen in the HOMSTRAD database of structurally derived sequence alignments [28]. Using the structure of the accepted wild type form of ovine PrP, the ALRQ allelic variant, an expected sequence substitution pattern for each amino acid position, given its structural environment, can be derived from the tables of the log-odds probability values. Accordingly, sequences homologous to ALRQ ovine PrP were identified by a BLASTp search [29] against the NCBI database, using the BLOSUM62 substitution matrix and an E-value threshold of 10^−6^. Mutant or incomplete PrP sequences were removed from the analysis. Alignment of homologous PrP amino acid sequences via the Crescendo algorithm (http://www.bioinf.manchester.ac.uk/crescendo) allowed the observed sequence substitution pattern of amino acids at every position in the polypeptide chain to be determined. The Kullback-Leibler conservation score, which is a measure of statistical similarity between the observed and expected sequence substitution distributions, was subsequently calculated [30]. This sequence conservation score identifies the residue positions that have a higher degree of observed sequence conservation than would be expected on the basis of the local structural environment. These additional restraints on allowed amino acid substitutions are either due to unusual structural requirements of the particular protein fold or to particular functions mediated by interactions with other molecules. Crescendo conservation scores associated with every amino acid residue was assigned to the three-dimensional coordinate of the atom most likely to be responsible for conservation of that particular amino acid. A three-dimensional Gaussian mask was placed at the position of the chosen coordinate and residue scores summed with scores from other residues if they were close in three dimensions (within the expanse of the mask). These summed mask scores were contoured and mapped onto the surface of the crystal structure of the ALRQ ovine PrP (PDB code 1TPX). The averaging, masking, and contouring were performed using the Kin3Cont component of the Kinemage suite of software (available at http://kinemage.biochem.duke.edu/).

### 2.2. Comparative Modelling

Models of the C-terminal domain (amino acid residues 110–228) of the ALRQ, AFRQ, and ALHQ variants of ovine PrP were built using the program MODELLER [31] using default parameters based upon the X-ray structure of ovine PrP (PDB code 1TPX) [32]. The region that comprised amino acid residues 112–121 was built as an *α*-helix of 3 turns based on secondary structure predictions using the prediction program Jpred [33].

### 2.3. Molecular Dynamics Simulations

The molecular dynamics simulations (*mds*) were carried out with the program Gromacs [34] using the OPLS-AA/L all-atom force field. A model of each allelic variant of ovine PrP was placed in an 80 × 80 × 80 Å box containing approximately 5500 water molecules and energy minimised for 1000 steps to remove any unfavourable contacts. Simulations were performed for each allele at 300 K for 15 ns at neutral pH (pH 7, above the pKa of histidine). Accordingly, glutamate and aspartate residues were negatively charged; lysine and arginine were positively charged and histidine residues were neutral. The simulations (*n* = 7 for each PrP variant) were carried out using 1 fs step size and the coordinates saved every 100 ps. Long-range electrostatic interactions were calculated using Particle Mesh Ewald.

### 2.4. Cloning, Expression, and Purification of Ovine Recombinant PrP

Expression constructs for mature length AFRQ and ALHQ ovine PrP (amino acid residues 25–232) were generated by site-directed mutagenesis of wild type ALRQ ovine PrP DNA (with methionine at residue 112) in a pET23b backbone [35]. Mutations were verified by DNA sequencing. Recombinant PrP was purified from BL21(DE3) pLysS* Escherichia coli* expressing ovine PrP in a method adapted from Hornemann et al. [7] and described in detail previously [36]. Oxidised and refolded recombinant PrP was stored at −80°C.

### 2.5. Anti-PrP Monoclonal Antibodies

The anti-PrP monoclonal antibodies FH11 [37] and V47 [38] have been described in detail previously. Monoclonal antibodies FH11 and V47 react with amino acid residues 54–58 and 217–232 of ovine PrP, respectively.

### 2.6. Metal-Ion Treatment of Ovine Recombinant PrP

Recombinant PrP (20 *μ*M) was dialysed into water at 4°C for 1 day with mild stirring before incubation with copper or manganese sulphate at 0.2 mM or 2.0 mM at 37°C for 20 hours. Samples were maintained at 4°C for a further 5 days prior to use in ELISA.

### 2.7. Sarkosyl Treatment of Ovine Recombinant PrP

20 *μ*M recombinant PrP in 50 mM sodium acetate (pH 5.0) was incubated with 0.005% or 0.008% (final concentration v/v) sarkosyl (prepared in MilliQ water) at 37°C for 1 hour. Reaction tubes were incubated at 4°C for 5 minutes. The samples were centrifuged at 13,000 ×g for 3–5 seconds and the supernatant analysed by CD spectroscopy.

### 2.8. Circular Dichroism Spectroscopy

Samples of recombinant PrP at concentrations of 25 *μ*M prepared in 50 mM sodium acetate were used for CD spectroscopic analysis. All samples were centrifuged at 13,000 ×g at 4°C for 30 minutes prior to analysis. CD spectra were recorded in a 0.5 mm path-length quartz cuvette at 20°C, under constant nitrogen flushing using a JASCO 810 spectropolarimeter. At least 10 spectra were accumulated and the values were expressed as molar ellipticity (*θ*). Secondary structure content was determined from deconvoluted CD spectroscopic data using the CDNN programme [39, 40].

### 2.9. ELISA

Direct ELISA: recombinant PrP protein samples at the desired concentration were coated onto 96-well flat-bottomed plates and incubated overnight at 4°C. Excess antigen was removed and the wells blocked with PBS containing 5% non-fat milk for 1 hour at 20°C. Plates were washed three times with PBS containing 0.1% Tween 80 (PBS-T). A 50 *μ*L volume of purified anti-PrP monoclonal antibody at 3 *μ*g/mL was added to the wells and the plates were incubated for 1 hour at 20°C, followed by three washes with PBS-T. Subsequently, 50 *μ*L of anti-mouse IgG-biotin conjugate (Sigma) was added to the wells at a dilution of 1 : 3000 and plates were incubated for 1 hour at 20°C, followed by three washes with PBS-T. The ELISA was completed as described below. Aggregation-specific ELISA: anti-PrP monoclonal antibody V47 was coated onto 96-well flat-bottomed plates at 1 *μ*g/well and incubated overnight at 4°C. Excess antibody was removed and the wells were blocked with PBS containing 1% fish gelatin for 2 hours at 20°C, followed by three washes with PBS-T. Recombinant PrP protein samples at the desired concentration were added to the wells and the plates were incubated for 1 hour at 20°C, followed by three washes with PBS-T. Biotinylated monoclonal antibody V47 at 1 *μ*g/mL was added to the wells for 1 hour at 20°C, followed by three washes with PBS-T. The ELISA was completed as described below. Colour development in both the* direct* and the* aggregation-specific ELISA* was achieved by the addition of 50 *μ*L of avidin-alkaline phosphatase conjugate (Sigma) at 1 : 2000 dilution and plates were incubated for 1 hour at 20°C. The plates were then washed three times in PBS-T and once with ELISA buffer (0.05 M glycine, 0.03 M NaOH, 0.25 mM MgCl_2_, and 0.25 mM ZnCl_2_) before addition of 50 *μ*L of the substrate* p*-nitrophenyl phosphate (Sigma) at 0.5 mg/mL in ELISA buffer for 30–60 minutes at 20°C. Absorbance was measured at 415 nm on a Bio-Rad 680 microplate reader.

### 2.10. Statistical Analysis

Statistical analyses of the data, where relevant, was performed using one-way ANOVA with Tukey HSD (honestly significant difference) for* post hoc* analysis or the two-tailed Student's *t*-test (unpaired samples) for analyses between genotypes using the Prism 4 software package (GraphPad).

### 2.11. Nomenclature

Amino acid residue numbers refer to the ovine PrP sequence unless stated otherwise.

## 3. Results

### 3.1. Sequence Conservation and Molecular Dynamics Simulations

The NMR structures of PrP from a variety of different mammalian species have now been described, as well as crystal structures of the globular domain of human and ovine PrP [6, 7, 9, 11, 32, 41–44]. In all of the species investigated so far, PrP consists of a flexible N-terminal region comprising ≈100 amino acids followed by a globular C-terminal region of ≈100 amino acids. The overall fold of the crystallised part of the C-terminal domain of ovine PrP is predominantly globular and contains 3 helices that comprised helix-1 (amino acid residues 146–158); helix-2 (amino acid residues 174–196); and helix-3 (amino acid residues 203–228) [32]. Helix-1 was flanked by *β*-strand-1 (amino acid residues 129–134) and *β*-strand-2 (amino acid residues 163–167) [32]. A stable disulphide bond was predicted between Cys182 and Cys217, which connected helix-2 and helix-3, while Asn184 and Asn200 were predicted to be N-linked glycosylation sites [32].

**Figure 1:**
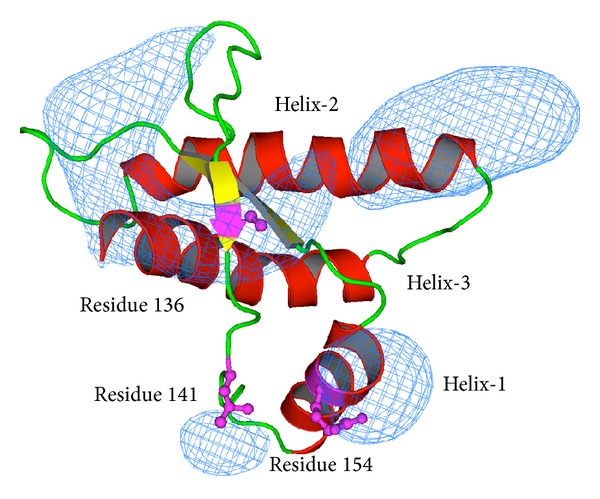
Location of clusters of high sequence conservation within the ovine PrP molecule. Sequence conservation scores for all the amino acids in PrP were mapped on the crystal structure of ALRQ ovine PrP and neighbouring residue scores contoured. The blue colouration highlights those areas with the greatest degree of sequence conservation.

In order to predict functional or critical structural amino acid residues that may contribute to the conformational change within PrP, we subjected the protein to computational analysis. We first analysed homologous PrP sequences from different species (a total of 284 sequences from 131 species) by the Crescendo method [27] to identify the degree of conservation of individual amino acids in the prion protein. Sequences homologous to ALRQ ovine PrP were identified by a BLASTp search [29] against the NCBI database. The data in Table 1 list amino acids within the PrP molecule that have a conservation score of >1, which is indicative of those residues more conserved than expected based upon its structure. This analysis showed that there are several regions within ovine PrP where the amino acid sequence was more conserved than expected. These regions were located around (i) amino acid Met157 in helix-1; (ii) amino acid His143; (iii) the loop between helix-2 and helix-3, which comprised amino acid residues 198–202; (iv) amino acid 136, with two conserved lobes, one centered upon Met132 in *β*-strand-1 and the other on Gln163 in *β*-strand-2. The locations of clusters of high sequence conservation within the ovine PrP molecule were contoured and mapped, where possible, onto the surface of the ALRQ variant and are shown in Figure 1.

**Figure 2:**
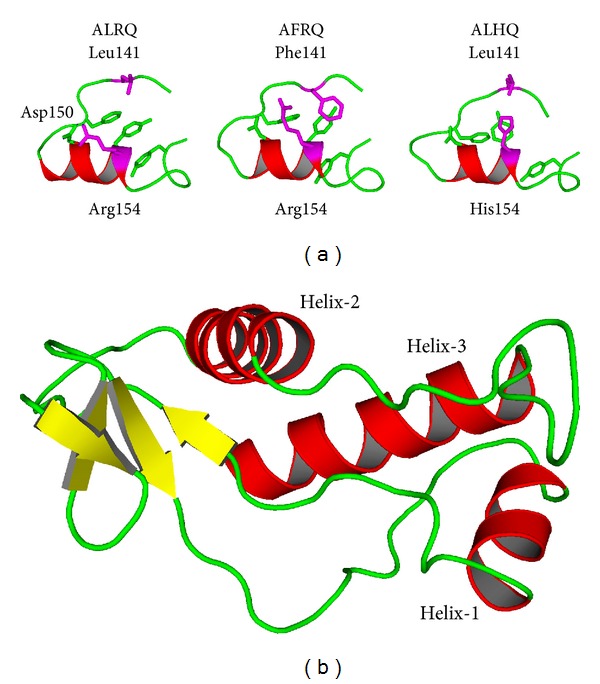
Ribbon diagrams that demonstrate structural features of ovine PrP. (a) Side-chain interactions in the vicinity of ovine PrP helix-1. Amino acid residue positions 141 and 154 are shown in magenta. Amino acid residue Arg154 that is present in the ALRQ allelic variant provides an extra interaction with Asp150. In contrast, the Phe141 in AFRQ and His154 in ALHQ interact with the aromatic stack that comprises amino acid residues Phe144, Tyr153, and Tyr160. (b) N-terminal *β*-sheet region of ALRQ. The structure of ALRQ forms an extended *β*-sheet comprising amino acid residues 112–121, *β*-strands 1 and 2 after* mds*.

We subsequently performed* mds* with models of ALRQ, AFRQ, and ALHQ ovine PrP in order to investigate how the polymorphisms at amino acid residues 141 and 154 affected the conformational variation of the conserved regions of the ovine prion protein. The region around the conserved amino acid Met157 of helix-1 was heavily influenced by genotypic variation at amino acid residues 141 and 154 of ovine PrP as shown in Figure 2(a). The charged amino acid residues within helix-1 formed many conserved side-chain interactions that stabilised its helical structure and orientation. These interactions include Glu149 with Asn146; Asp147 with Arg151 and Glu155; His143 with Arg231. In the ALRQ variant, there were additional interactions that involved the solvent exposed Arg154 with the side-chain of Asp150 and the backbone of Leu142. However, in the AFRQ genotype, the latter interactions were rarely seen. The Phe141 formed an extended aromatic-stacking interaction with Phe144, Tyr153, and Tyr160. Similarly, in ALHQ ovine PrP, His154 also formed extended aromatic-stacking interactions with Phe144 and Tyr153. These different interactions in the vicinity of helix-1 subsequently have an effect on the structure and secondary structure content of other regions of the C-terminal domain of ovine PrP, in particular helix-2. Important interactions that normally maintain the structure of the last turn of helix-2 involve the side-chains of Gln189, Thr193, Thr194, Thr195, and Lys197, which are conserved amino acids, highlighted by the Crescendo analysis.

The loop between helix-2 and helix-3 was influenced by the helix-2 secondary structure. Helix-2 was unwound at its C-terminus by up to two turns in the ALRQ allelic variant and this unwinding occurred to a lesser extent and less frequently in ALHQ and AFRQ ovine PrP. A key interaction seen in all three allelic variants of ovine PrP occurred between the highly conserved amino acid His190 in helix-2 and the backbone of Arg159 in the loop between helix-1 and *β*-strand-2, which limits the unwinding of helix-2. This interaction would be lost at low pH allowing for more unwinding of helix-2 and a greater conformational change, especially in ALRQ ovine PrP, as we have previously suggested [45]. This extensive unwinding of helix-2 at low pH has also been shown for Syrian hamster PrP (amino acid residues 90–231), which also possessed an Arg residue at the equivalent codon to ovine amino acid residue 154 [46].

Amino acid residues 116–123 of ovine PrP comprise a palindromic sequence (AGAAAAGA) that is present in a part of ovine PrP that has been considered as disordered [32]. Significantly, our* mds* analysis reported here showed that the region around the palindromic sequence and the conserved region around amino acid residue 136 of ovine PrP underwent structural changes that were influenced by polymorphisms at amino acids 141 and 154. In the ALRQ variant, the helical region comprising amino acid residues 112–121, present at the start of the* mds*, unwound completely and resulted in the formation of additional *β*-strands, forming an extended *β*-sheet with *β*-strands 1 and 2 as shown in Figure 2(b). This helical unwinding and increase in *β*-sheet content occurred most frequently in the ALRQ variant (28%) and least frequently in ALHQ ovine PrP (18%). A network of interactions that stabilised the formation of these additional *β*-strands occurred between several of the amino acid residues highlighted by the Crescendo analysis including His114, Ala136, Gln163, Gln215, Thr219, and Gln220. While the N-terminal region of PrP has been regarded as a disordered protein domain [47, 48], it paradoxically influences structure in the remainder of the protein [49]. Here we found that the formation of the extended *β*-sheet structure in the N-terminal region of ovine PrP correlated with the structural changes in the C-terminal domain of the protein. Specifically, the last two turns of helix-3 (from Gln220) were found to unwind and move towards helix-2. These C-terminal structural changes allowed the formation of an extensive network of interactions between amino acid residues Tyr221, Arg223, and Glu224 of helix-3 and the backbone of amino acid residues 160, 170, 172, and 173 within the *β*-strand 2-helix-1 loop.

### 3.2. Generation of Ovine Recombinant PrP

Collectively, our computational analysis has shown that genotypic variation at amino acid residues 141 and 154 within ovine PrP has the potential to induce local and long-range effects upon conserved regions of amino acid sequence in the protein that are likely to regulate its structure and therefore its conformational change. In order to attempt to validate this computational analysis we performed structural studies with ALRQ, AFRQ, and ALHQ ovine recombinant PrP. These polymorphic variants of ovine PrP were produced from a pET23b-mediated prokaryotic expression system [35]. Consistent with the expected secondary structure all three PrP isoforms produced CD spectra indicative of an *α*-helical enriched protein at pH 5.0 (data not shown).

### 3.3. Sarkosyl Induces Conformational Changes in Ovine Recombinant PrP

**Figure 3:**
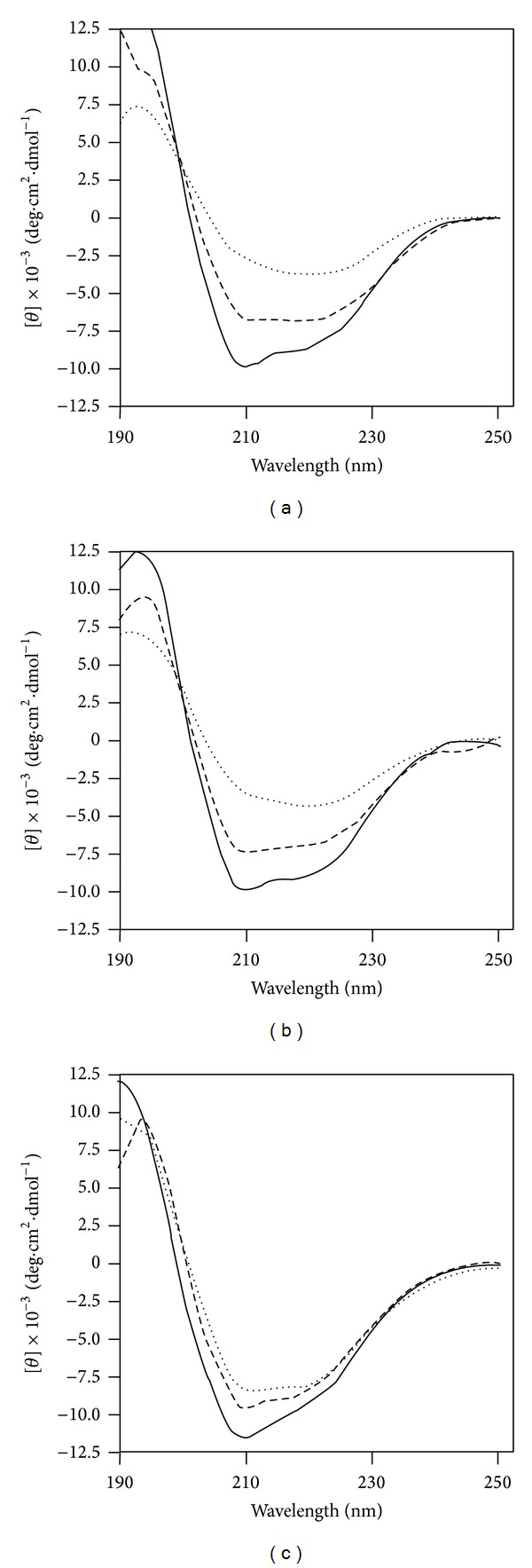
CD spectra of sarkosyl-treated ovine recombinant PrP. Conformational changes of (a) ALRQ; (b) AFRQ; or (c) ALHQ ovine recombinant PrP following treatment with water (continuous line); 0.005% sarkosyl (large dashed line); or 0.008% sarkosyl (small dashed line). Data shown are representative CD spectra for different batches of ovine recombinant PrP ALRQ (*n* = 5); AFRQ (*n* = 1); and ALHQ (*n* = 3).

Anionic detergents such as sarkosyl have been reported to induce *β*-sheet conformation in PrP leading to amorphous and fibrillar aggregation [50, 51]. The impact of polymorphisms in ovine PrP on sarkosyl-induced conformational changes was investigated by CD spectroscopy. Following exposure to sarkosyl the CD spectroscopic profiles of all three ovine PrP variants showed changes indicative of an increase in the proportion of *β*-sheet secondary structure as shown in Figure 3. Secondary structure analysis of the profiles, using the CDNN algorithm, indicated that the *β*-sheet content of ALRQ PrP increased from 21.7 ± 1.0% in the absence of sarkosyl to 34.8 ± 4.2% after exposure to 0.008% sarkosyl; AFRQ PrP showed an increase from 22.5% to 35.9%; and ALHQ PrP showed an increase from 23.3 ± 0.7% to 28.2 ± 2.4%. The relative percentage increase of *β*-sheet content following exposure to 0.008% sarkosyl was approximately 60% in ALRQ and AFRQ but only 21% in ALHQ ovine PrP. To further quantify these changes we measured the molar ellipticity of ovine PrP at the maxima and minima wavelengths characteristic of *α*-helical proteins (193 nm, 208 nm, and 222 nm) and those with well-defined antiparallel *β*-pleated sheets (195 nm and 218 nm). After exposure to sarkosyl, ALRQ ovine PrP demonstrated changes in molar ellipticity values that were consistent with a loss of *α*-helical secondary structure and an increase in *β*-sheet structure as shown by the data in Table 2. The same trend was shown by both AFRQ (data not shown) and ALHQ ovine PrP. The absolute changes in molar ellipticity values for sarkosyl-treated ALRQ ovine PrP were significantly greater in both extent and magnitude than the similarly treated ALHQ allelic variant (*P* < 0.05 at the stated values).

### 3.4. ELISA Measurement of Secondary Structural Changes in Cu^2+^ Treated PrP

**Figure 4:**
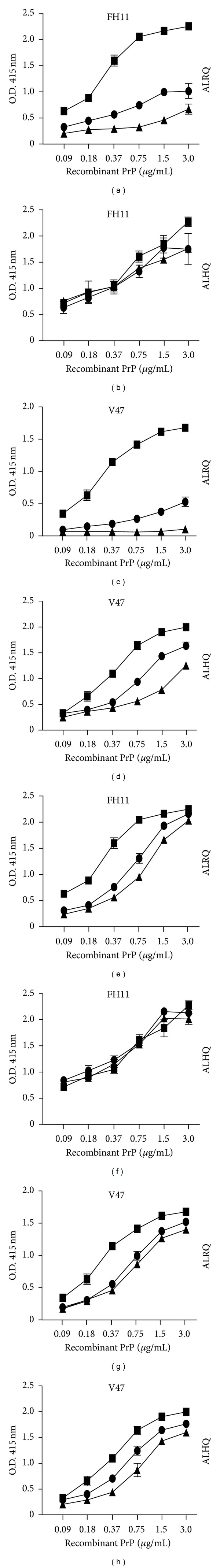
ELISA reactivity of metal ion-treated ovine recombinant PrP. Ovine recombinant PrP ALRQ (a, c, e, g) or ALHQ (b, d, f, h) was treated with water (black square) or either 0.2 mM (black circle) or 2 mM (black triangle) copper (a–d) or manganese (e–h) at 37°C as described in Section 2. Serial 2-fold dilutions of recombinant PrP were analysed by direct ELISA for reactivity with anti-PrP monoclonal antibody FH11 (a, b, e, f) or V47 (c, d, g, h). The data shown are the means of triplicate wells ± SD.

The conformational changes in ovine PrP that result in a decrease in *α*-helical content and an increase in *β*-sheet structure may reflect a change in epitope exposure within the prion protein. To assess this, ovine PrP secondary structural changes induced by Cu^2+^ treatment were analysed by ELISA using anti-PrP monoclonal antibodies as shown in Figure 4. Treatment with either 0.2 or 2 mM Cu^2+^, reduced the reactivity of ALRQ PrP with the N-terminal specific anti-PrP monoclonal antibody FH11 by >50% (Figure 4(a)) but did not alter the reactivity of ALHQ (Figure 4(b)). Reactivity to the C-terminal-specific monoclonal antibody V47 was reduced to a greater extent for ALRQ (Figure 4(c)) than for ALHQ PrP (Figure 4(d)). The response of Cu^2+^-treated AFRQ PrP to FH11 was intermediate to that seen for ALRQ and ALHQ, whilst that to V47 was similar to that of ALRQ (data not shown). These conformational changes in PrP were specific for Cu^2+^ as treatment with Mn^2+^ produced much more limited changes in conformation (Figures 4(e)–4(h)).

### 3.5. Genotypic Differences in Ovine PrP Aggregation

We investigated the potential of different genotypes of ovine PrP to aggregate through the use of an aggregation-specific ELISA. This immunoassay was designed on the rationale that PrP aggregates might be expected to display multiple copies of an epitope recognised by an anti-PrP monoclonal antibody while monomeric PrP protein will have only one copy of the epitope exposed [52]. Consequently, we used the same anti-PrP monoclonal antibody to detect aggregated PrP molecules, unlike a conventional capture-detector ELISA that utilises two different monoclonal antibodies.

**Figure 5:**
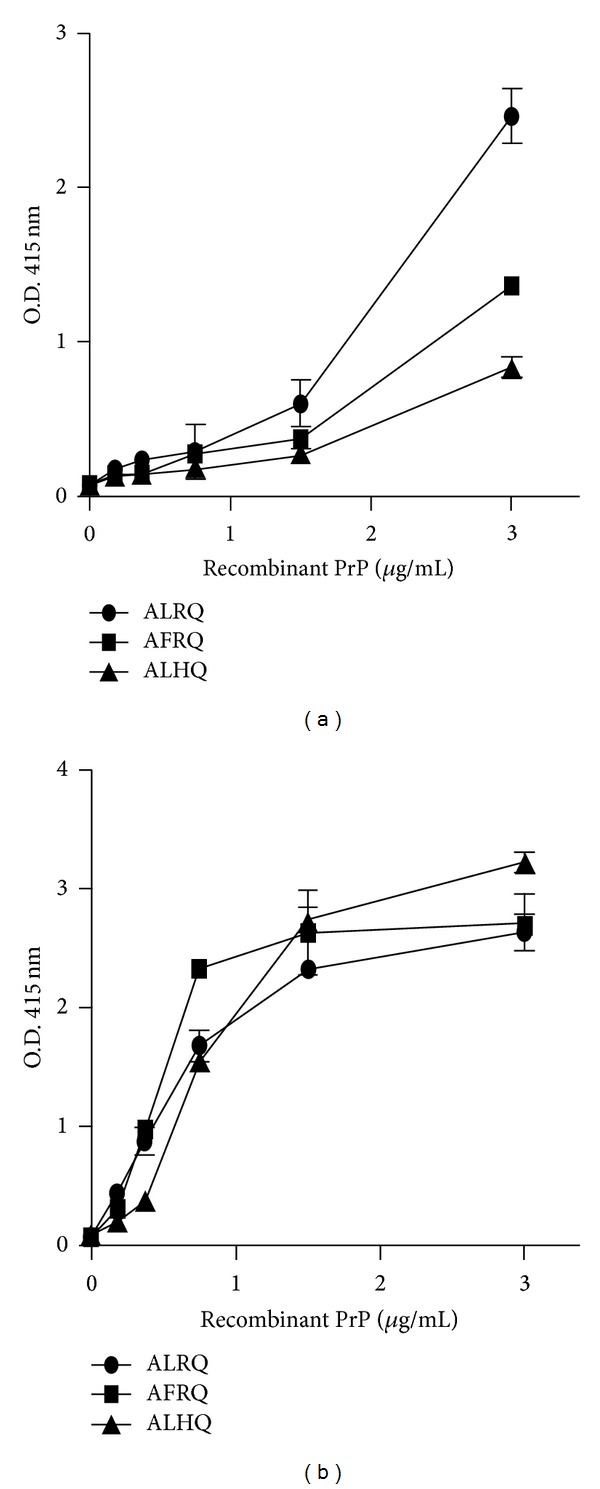
Aggregation-specific ELISA reactivity of ovine recombinant PrP. Ovine recombinant PrP ALRQ (black circle), AFRQ (black square), and ALHQ (black triangle) aged 12 months was analysed by (a) aggregation-specific ELISA and (b) direct ELISA using anti-PrP monoclonal antibody V47 as described in Section 2. Serial 2-fold dilutions of recombinant PrP were analysed as shown. The data shown are the means of triplicate wells ± SD.

The data in Figure 5(a) show the reactivity of different allelic variants of ovine PrP in the aggregation-specific ELISA that utilised anti-PrP monoclonal antibody V47, whereby an increase in reactivity is indicative of PrP aggregation. The order of reactivity in this ELISA for ovine PrP that had been aged for 12 months was ALRQ > AFRQ > ALHQ. When ovine PrP was aged for 18 months and then assessed by the aggregation-specific ELISA, the level of reactivity of the ALRQ variant was decreased, while the level of reactivity seen by AFRQ and ALHQ PrP was increased compared with the equivalent genotype of PrP aged for 12 months (data not shown). As a control, the total level of age-matched PrP protein was assessed by direct ELISA using anti-PrP monoclonal antibody V47. The data in Figure 5(b) show that the different allelic variants of ovine PrP showed a similar reactivity in the direct ELISA, which confirmed that similar levels of PrP protein were present in each sample.

## 4. Discussion

A fundamental event in the pathogenesis of prion diseases, such as scrapie of sheep, is the misfolding of PrPC and the accumulation of PrPSc. It is important therefore to study PrP structural changes so as to understand the molecular mechanisms of misfolding and aggregation and how these processes may be regulated. Here we have investigated how genotypic variation at amino acid residues 141 and 154, which are associated with susceptibility to atypical scrapie, influence conformational variation in conserved regions of the ovine prion protein.

We utilised the Crescendo method to identify those amino acid residues in ovine PrP with a higher degree of conservation than expected on the basis of the local structural environment and they were therefore considered to be evolutionary conserved. All of the identified amino acid residues were solvent exposed and their positions within PrP were spread across most regions of the protein. The most conserved region, and also that with the largest exposed surface, was found to be the loop between helix-2 and helix-3, which comprised amino acid residues 198–202. We have previously identified this as a region of genotypic structural variation with regard to the ALRR, ALRQ, and VLRQ allelic forms of ovine PrP [26]. This region has been identified as one of potential structural importance in the conversion of PrPC to PrPSc by NMR studies that show perturbation of the helix-2-helix-3 loop in denaturant-induced folding intermediates [53]. Furthermore, mutations in this region are associated with distinct types of human prion disease. For example, the mutation F198S in human PrP is associated with a familial form of Gerstmann-Sträussler-Scheinker (GSS) disease [54], whilst the E200 K mutation is associated with familial CJD [55]. A second region displayed two conserved lobes, one centered upon Gln163 in *β*-strand-2 and the other on Met132 in *β*-strand-1 that flanks amino acid residue Ala136. The mutation of Ala136Val in ovine ALRQ PrP gives rise to the VLRQ allelic variant that is associated with high susceptibility to classical scrapie disease of sheep [17]. In addition, this conservation cluster lies within that segment of PrP that displays a high structural plasticity [56–59]. Two other regions of PrP, one centered on amino acid residue Met157 in helix-1 and nearby amino acid residue His143, showed significant sequence conservation. These are close to the sites of polymorphisms associated with atypical scrapie-susceptible genotypes, namely, amino acid residues 141 and 154. Collectively, PrP sequence conservation analysis has shown that, in addition to the well-established scrapie-susceptibility associations of amino acid residues 136 and 171, Met132 at the start of *β*-strand-1; His143; Met157 in helix-1; and the helix-2-helix-3 loop region may be required for the structural stability of the prion protein or are involved in hitherto unidentified molecular interactions, for example, oligomeric formation.

In common with other species forms of PrP, ovine PrP contains a predominantly globular C-terminal domain and a less structured N-terminal region [6, 8–11, 32, 41–44]. We have previously shown that* mds* can provide good agreement between modelled structures of ovine PrP and those derived by experimental studies [26]. Here we have used* mds* to model amino acid residues 110–228 of ovine PrP in order to determine the effect of polymorphisms in the *β*-strand 2-helix-1 loop on the conserved regions of this protein. Our* mds* analysis showed that while the general features of the PrP fold were retained, as seen for previous experimentally derived structures of ovine PrP [32, 44], significant differences were evident in the formation and interaction of secondary structural elements within the different prion protein genotypes. Significantly, we have found that the region comprising amino acid residues 112–124, which includes the palindromic sequence comprising amino acids AGAAAAGA, was able to form additional *β*-strands that resulted in the formation of an extended antiparallel *β*-sheet arrangement in combination with *β*-strands 1 and 2. This increase in *β*-sheet content also showed genotype variation, occurring most frequently in ALRQ and least frequently in ALHQ ovine PrP. The region around the palindromic sequence PrP has been predicted to form *β*-strands [60] but inherent disorder has hampered determination of its structure. Experimental evidence for the formation of an extended *β*-sheet arrangement in this N-terminal region has been achieved by the recent cocrystalisation of human PrP with an anti-PrP monoclonal antibody [61]. While the N-terminal region of PrP has been regarded as a disordered protein domain [47, 48], our observation here and similar observations by others [60, 61], of the formation of an extended *β*-sheet arrangement in the vicinity of the palindromic sequence, suggests that the N-terminal region of PrP has more structure than previously proposed. Alternatively, this region of the N-terminal domain of PrP may serve as a site that mediates *β*-sheet enrichment during the formation of disease associated PrP. It has been shown that peptide fragments of PrP that contain the palindromic sequence show a high tendency to aggregate into *β*-sheet-rich amyloid fibrils [62] and are neurotoxic [63].

The N-terminal region of PrP may play a role in modulation of PrP aggregation since this region influences the amount of secondary structure in the remainder of the molecule [49]. Our* mds* analysis reported here has shown that formation of the extended *β*-sheet structure in the N-terminal region of ovine PrP correlated with significant structural changes in the C-terminal region of the protein. The conserved amino acid residue Met157 of helix-1 was heavily influenced by genotypic variation at amino acid residues 141 and 154 of ovine PrP. Differences in the interactions in this region were subsequently found to have an effect on the secondary structure content of other regions of the C-terminal domain of ovine PrP, in particular helix-2. Unwinding of helix-2 occurred to a lesser extent and less frequently in the ALHQ and AFRQ ovine PrP compared to that seen in the ALRQ allelic variant as shown here and in our previous* mds* studies [26]. The last two turns of helix-3 (from Gln220) were found to unwind, which is seen more frequently in the ALRQ genotype. Once fully unwound, the end of helix-3 was able to interact with the *β*-strand 2-helix-2 loop, which adopted a similar conformation as seen in the other examples of PrP that display an N-terminal extended *β*-sheet structure [60, 61]. These C-terminal structural changes in ovine PrP allowed the formation of an extensive network of interactions between amino acid residues Tyr221, Arg223, and Glu224 of helix-3 and the backbone of amino acid residues 160, 170, 172, and 173 within the *β*-strand 2-helix-2 loop. Structural flexibility of the *β*-strand 2-helix-2 loop region and helix-3 has been highlighted as crucial determinants for susceptibility to prion disease [60, 64, 65]. Collectively, our computational analysis has shown that genotypic variation at amino acid residues 141 and 154 within ovine PrP has the potential to induce long-range effects upon conserved regions of amino acid sequence in the protein that are likely to regulate its structure and therefore affect its conformational change. We supported our computational structural analysis of allelic variants of ovine PrP with experimental studies using detergent- or copper-induced conformational change in recombinant ovine PrP.

The anionic detergent sarkosyl is widely used in PrPSc purification and enhances extraction efficiency of PrPSc and stimulates the PrPSc-induced conversion reaction [66, 67]. Treatment of allelic variants of ovine recombinant PrP with sarkosyl resulted in an increase in the proportion of *β*-sheet secondary structure that was of the order ALRQ ≥ AFRQ > ALHQ. This suggests that amino acid residue 154 of ovine PrP, and the polymorphisms at this position, contributes to the susceptibility of anionic detergent-mediated conformational change. Previously, it has been proposed that sarkosyl induces conformational change by inserting detergent into the hydrophobic core of PrP, thereby disrupting its conformation and facilitating hydrophobic protein-protein interactions [51]. Since ALHQ has been reported to have a less exposed hydrophobic core than other allelic variants of ovine PrP, such as VLRQ [68], the hydrophobic interactions between detergent micelles and the core of ALHQ may not be as extensive as that seen with other ovine PrP variants.

The N-terminal region of PrP contains high affinity binding sites for Cu^2+^ ions and acquires structure following copper binding [69, 70]. This N-terminal conformational change may modulate the propensity of PrP aggregation since this region influences the amount of secondary structure in the remainder of the molecule [49]. As a consequence of binding Cu^2+^ ions, PrP undergoes conformational changes that involve interactions between different regions of the protein [5, 36, 71, 72]. For example, amino acid residues close to the C-terminal region of *α*-helix-1 and the nearby loop between *β*-strand-1 and *α*-helix-1 interact with Cu^2+^ coordination groups in the N-terminal region of the protein [73]. We have previously found that the Met112Thr polymorphism in ovine ALRQ PrP, located within a Cu^2+^-binding site in the N-terminal region of the protein [53, 74, 75], affects copper-induced structural changes within the C-terminal region of the prion protein [35]. Here, we have shown by capture-detector ELISA that the structural variation induced by Cu^2+^ within ALRQ ovine PrP was associated with conformational changes in the N-terminal and C-terminal regions of the protein but principally only in the C-terminal region of the ALHQ variant. The modulation of epitope exposure in the N-terminal region of ALRQ ovine PrP, determined by ELISA, correlated with its enhanced tendency for formation of additional *β*-strands in this region of the protein compared to the ALHQ variant, as shown in the* mds* analysis. The enhanced detergent- and copper-induced structural changes displayed by ALRQ ovine PrP corresponded with an increased capacity of this genotype of ovine PrP to undergo age-induced multimerisation as measured by the aggregation-specific ELISA. However, while the ALRQ variant showed the greatest tendency for aggregation amongst the different polymorphic forms of ovine PrP tested here, after an extended period of aging its reactivity was decreased below that of similar aged AFRQ and ALHQ PrP. We speculate that the time-dependent decrease in reactivity of ALRQ ovine PrP in the aggregation-specific ELISA was a consequence of its greater propensity to aggregate, compared to the AFRQ and ALHQ variants, which resulted in previously exposed epitopes subsequently becoming obscured. This view is supported by our previous observation that denatured ALRQ ovine PrP undergoes a more rapid rate of aggregation compared to the ALHQ variant [45].

The conformational properties of PrP are strongly pH-dependent [76] and various in vitro studies have reported a relationship between pH and misfolding and aggregation of PrP [77]. However, the exact subcellular location and therefore the pH of the environment where this process may occur have not been fully defined. Here we have collectively studied conformational variation in ovine PrP genotypes at neutral and acidic pH. Neutral pH conditions are characteristic of those at the plasma membrane where PrPSc has been reported to form soon after prion exposure of cells in vitro, even under conditions that prohibit endocytic activity [78]. Acidic pH conditions are characteristic of the intracellular endosomal compartments [79] where PrPC is sequestered during its internalisation. Reports using in vitro cell culture models have proposed that PrPC misfolding and accumulation may occur during endocytosis in both late and recycling endosomes [80–82]. Here we have found that the same trend in genotypic conformational variation in ovine PrP, namely, ALRQ > AFRQ > ALHQ, was seen under both acidic and neutral pH conditions.

Transmission of classical scrapie is aided by shedding and secretion of infectious prions into the environment from affected animals [83]. It is reasonable to speculate that the conformational properties displayed by ALRQ ovine PrP may be a prerequisite for the formation of naturally transmissible ovine prions, which presumably require a certain level of stability to withstand the transmission process. This would appear to be the case since allelic variants of ovine PrP associated with classical scrapie have a greater efficiency for in vitro conversion into a protease-resistant form following interaction with PrPSc [16, 84]. Furthermore, the PrPSc associated with classical scrapie is more resistant to proteolytic digest than its counterpart in atypical scrapie [16, 84]. The distinct biochemical and biophysical signatures, shown by different allelic variants of ovine PrP [16, 36, 84, 85], appear to be underpinned by their ability to undergo conformational change in key conserved regions of the protein. Using a combined computational and experimental approach, we have shown that this conformational change can be influenced by single amino acid mutations at amino acid residue 141 located in the loop following *β*-strand-1 and amino acid residue 154 located in *α*-helix-1. For example, ALRQ ovine PrP, which is associated with classical scrapie, showed an increased propensity to acquire increased *β*-sheet content and to aggregate compared to ALHQ PrP, which is associated with atypical scrapie. These data suggest a structural correlation for genotypic variants of ovine PrP and their association with different forms of scrapie.

## Figures and Tables

**Table 1:** Sequence conservation scores for amino acid residues in ovine PrP predicted by the Crescendo algorithm. Ovine PrP amino acid residues with conservation scores >1 are listed. The conservation score quantifies the degree of sequence conservation at an alignment position compared to the average sequence conservation. A score of >1 means the sequence conservation is more conserved than that expected based upon its structure.

Conservation score	Amino acid residue	Structural position
2.28	Met157	Helix-1
2.13	Gln163	*β*-strand-2
2.08	Met 132	*β*-strand-1
1.80	Thr194	C-terminus of helix-2
1.73	Thr195	C-terminus of helix-2
1.70	Thr191	C-terminus of helix-2
1.70	Thr219	Helix-3
1.67	Asn176	Helix-2
1.47	His143	*β*-strand-1-helix-1 loop
1.40	Gln220	Helix-3
1.38	His190	C-terminus of helix-2
1.38	His114	N-terminal domain
1.37	Gln215	Helix-3
1.35	Gln175	Helix-2
1.35	Thr204	Helix-3
1.21	Asn156	Helix-1
1.01	Ala136	*β*-strand-1

**Table 2:** Quantitative changes in molar ellipticity values of sarkosyl-treated ovine recombinant PrP. Batches of ovine recombinant PrP ALRQ (*n* = 5) and ALHQ (*n* = 3) were analysed by CD spectroscopy following treatment with sarkosyl as described in Section 2. Results are mean ± standard deviation molar ellipticity values at wavelengths characteristic for *α*-helical (193 nm, 208 nm, and 222 nm) and *β*-sheet (195 nm and 218 nm) proteins. Figures in brackets show percentage change in molar ellipticity values between sarkosyl- and water-treated samples. Statistical analyses of the data for individual PrP genotypes were performed using one-way ANOVA with Tukey HSD (honestly significant difference) for *post hoc* analysis or the two-tailed Student's *t*-test (unpaired samples) for analyses between genotypes using the Prism 4 software package (GraphPad).

Ovine PrP	Sarkosyl	Molar ellipticity (deg*·*cm^2^ *·*dmol^−1^) × 10^−3^				
		193 nm	208 nm	222 nm	195 nm	218 nm
ALRQ	None	14032 ± 2978	−9944 ± 642	−8879 ± 694	11779 ± 2205	−9624 ± 853
	0.005%	10530 ± 2176 (25%)	−6489 ± 1077∗ (35%)	−7195 ± 486∗ (19%)	9102 ± 1886 (23%)	−7562 ± 716∗ (21%)
	0.008%	7708 ± 2012∗ (45%)	−3576 ± 1311∗ (64%)	−4706 ± 1077∗ (47%)	6369 ± 1106∗ (46%)	−4819 ± 1354∗ (50%)

ALHQ	None	12690 ± 1952	−9988 ± 1713	−8703 ± 551	10993 ± 3150	−9643 ± 695
	0.005%	10454 ± 1594 (18%)	−7506 ± 1290 (25%)	−8274 ± 662^†^ (5%)	10131 ± 1882 (8%)	−8969 ± 448^†^ (7%)
	0.008%	10833 ± 3237 (15%)	−5315 ± 1962∗ (47%)	−7786 ± 831^†^ (11%)	9595 ± 1704^†^ (13%)	−7945 ± 602^∗†^ (18%)

*P < 0.05 for sarkosyl treatment in comparison with no sarkosyl treatment.

^†^ *P* < 0.05 for ALRQ versus ALHQ.
